# The obstetric care subsidy policy in Burkina Faso: what are the effects after five years of implementation? Findings of a complex evaluation

**DOI:** 10.1186/s12884-016-0875-2

**Published:** 2016-04-21

**Authors:** Rasmané Ganaba, Patrick G. C. Ilboudo, Jenny A. Cresswell, Maurice Yaogo, Cheick Omar Diallo, Fabienne Richard, Nadia Cunden, Veronique Filippi, Sophie Witter

**Affiliations:** AFRICSanté, Bobo-Dioulasso, Burkina Faso; London School of Hygiene and Tropical Medicine, London, UK; Centre Muraz, Bobo-Dioulasso, Burkina Faso; Institute of Tropical Medicine, Antwerp, Belgium; University of Aberdeen, Scotland, UK; Institute for Global Health and Development, Queen Margaret University, Scotland, UK

**Keywords:** Maternal health, Health system, Policy, Removal of user fees, Evaluation, Cost of obstetric care, Quality of care, Burkina Faso

## Abstract

**Background:**

Burkina Faso, like many low and middle income countries, has been taking a range of actions to address its poor maternal and neonatal health indicators. In 2006 the government introduced an innovative national subsidy scheme for deliveries and emergency obstetric care in public facilities. This article reports on a complex evaluation of this policy, carried out 5 years after its introduction, which examined its effects on utilisation, quality of care, equity and the health system as a whole, as well as its cost and sustainability.

**Methods:**

The evaluation was carried out in six purposively selected districts, as well as at national level, using a case study approach. Data sources included: national and district routine and survey data, household interviews with women who had recently given birth, data extraction from hospital and medical records, and key informant and health worker interviews.

**Results:**

The underlying secular trend of a 1 % annual increase in the facility-based delivery rate (1988–2010) was augmented by an additional 4 % annual increase from 2007 onwards (after the policy was introduced), especially in rural areas and amongst women from poor households. The absence of baseline quality of care data made it difficult to assess the impact of the policy on quality of care, but hospitals with the best level of implementation of the subsidy offered higher quality of care (as measured by health care near-misses), so there is no evidence of a negative impact on quality (as is often feared). Similarly, there is little evidence of unintended negative effects on untargeted services. Household payments for facility-based deliveries have reduced significantly, compared with payments before the policy, and the policy as a whole is affordable, costing about 2 % of total public health expenditure.

Concerns include that the amounts paid by households are higher than the rates set by the policy, and also that 7 % of households still say that they cannot afford to pay. Wealthier women have higher utilisation of services, as before, and the policy of fully exempting indigents is not being put into practice.

**Conclusions:**

These findings highlight the importance of maintaining the subsidy policy, given the evidence of positive outcomes, but they also point out areas where attention is needed to ensure the poor and most vulnerable population benefit fully from the policy.

## Background

Burkina Faso is a low-income country in West Africa with high maternal and child mortality (a maternal mortality ratio of 400 and under-five mortality rate of 102) [1, 2]. In order to accelerate progress towards Millennium Development Goals 4 and 5, Burkina Faso, along with many other sub-Saharan countries, launched a national subsidy policy which aimed at reducing user fees for maternity services. This subsidy policy was introduced in 2006 and covers all public health facilities and some private not-for-profit structures. The policy consists of a partial exemption of direct health care costs (80 % for all emergency obstetric care – EmOC - including transport in case of referrals, 80 % of uncomplicated deliveries in districts hospitals and health centres and 60 % of uncomplicated deliveries in regional and national hospitals), the remaining portion to be borne by patients. Burkina Faso’s subsidy policy goals were threefold: 1) reducing the costs of facility-based delivery care to women and their families, 2) enhancing the quality of facility-based delivery services, and 3) increasing women’s access to hospital facilities for complicated deliveries [3].

The policy implementation started in October 2006 in hospitals and in health centresfrom January 2007 [4]. The first evaluation was conducted from November 2008 to April 2009, which was too early to detect an impact [4]. The second study, which happened more recently, was conducted in only two districts out of 63, making it difficult to draw generalizable conclusions for the national health system [5]. In addition, all these studies were only conducted in districts with primary facilities (health centres and district hospitals) but without referral hospitals. Finally, neither study investigated the impact of the policy on quality of care and on the local health system. The present study aimed at filling these gaps of knowledge. Our study is a part of a multi-county research involving three sub-Saharan countries (Benin, Burkina Faso, Mali) and Morocco [6].

The overall study objective was to evaluate the policy of obstetric care subsidy in Burkina Faso. Thespecific objectives were to:

1. Determine if the introduction of the policy was followed by an increase of health services use, including facility-based deliveries and caesarean sections, and if the policy increased equity of access to health care;
2. Analyze the costs incurred by households during childbirth and collect the perceptions of people on the quality of services;
3. Evaluate the effects of the policy on the health system at district level (including both targeted and non-target health services) by 1) examining potential changes induced by the introduction of the policy on the work patterns and motivation of health workers, and 2) assessing the financing, financial effects on facilities and sustainability of the policy; and 3) examining trends in selected untargeted health services in the study districts;
4. Understand the effects of the policy on severe maternal and neonatal morbidity and on quality of care.

## Methods

### Study design

The study undertook a complex evaluation using a realist evaluation approach [7]. The complexity of the evaluation relates to the dynamic and non-linear nature of the policy intervention being studied. The group began by formulating a conceptual framework, which helped to define the key relationships for which data should be sought [7]. The research tools were designed around trying to measure changes in key nodes, as far as was possible given that baseline data for key variables was often not available and policies were introduced nationally (so without control groups). The nodes were fields within the theory of change which were predicted to be important in influencing the effectiveness of the policy (for example, changes to staff motivation, or to the costs of targeted services for women). Fixed mixed methods were used to gather data, using a convergent parallel design, with research conducted concurrently and triangulated in the synthesis phase [8]. Data were then integrated to answer the key evaluation questions [7]. Theevaluation in Burkina Faso was carried out in six purposively selected districts, as well as at national level, using a case study approach [8]. Each district presents a case study which can be compared with others, not only within the country but also across the four study countries.

For further details on the overall study, please see the FEMHealth protocol and overall findings, as well as the study protocol for Burkina Faso (which contains the research tools) and the full country report of findings [6, 9–11].

### Selection of study sites

As one of the objectives was to determine the effect of the policy on health service use including caesareans, eligible districts were chosen from amongst those which performed at least 50 caesarean sections per year before the start of the policy. Fewer than 50 caesareans per year can be a sign of severe dysfunction of the surgical theatre (no surgeon, no equipment) and could be a confounding factor when evaluating the impact of the abolition of user fees.

**Fig. 1:**
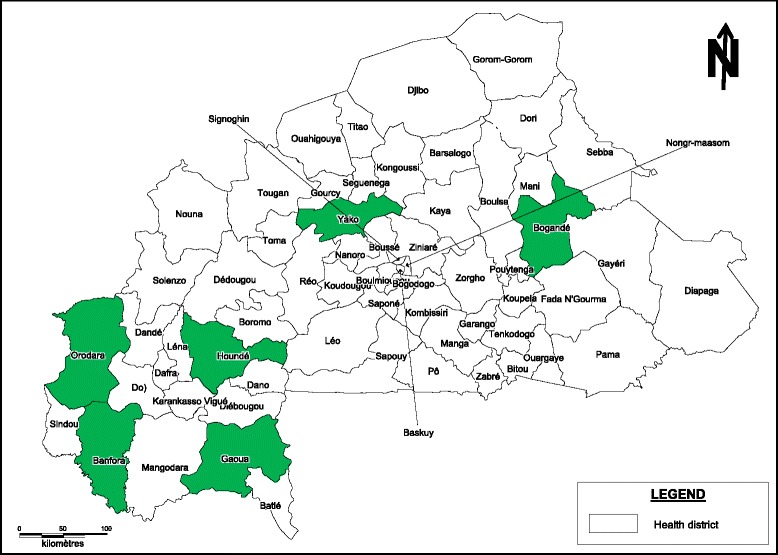
Map of the districts sampled to evaluate the obstetric care subsidy policy in Burkina Faso, Femhealth project 2011–2013. Footnote: we acknowledge the GIS service of Centre Muraz, MoH

In order to include a range of representative districts, with respect to health facilities’ accessibility (geographical and economic) and service supply and use, the districts were first classified based on four characteristics: caesarean rate, proportion of assisted deliveries, average distances to a health facility, and poverty rate [12]. Out of the 63 health districtsin Burkina Faso, data before the policy implementation were available in 52 districts (because 11 districts were newly created). Out of the 52 districts, 24 performed more than 50 caesarians per year before the policy implementation. The classification yielded four groups of which one was composed of seven districts all performing less than 50 caesarians per year before the policy. Thus, the study districts were selected from the three other groups. Two districts were selected within each group, giving a total of six districts. Four districts (Bogandé, Houndé, Orodara, Yako) with a district hospital (1st level of care) were selected out of 15 eligible and two districts (Banfora, Gaoua) with a regional hospital (2nd level of care) were selected out of nine eligible (Fig. 1). The study sites included five of the 13 health regions in the country. See Table 1 for a summary of the groupings.

**Table 1:** Summary of district groupings and site selection

	Caesarean rate	% assisted deliveries	Average distance to a health facility	Poverty rate	Description
Group 1	0,17	57,39	4,29	29,94	Districts with limited or non functional surgery; women go to the university hospital (central region)
Group 2	1,46	47,33	6,69	38,90	Medium utilisation; medium access and fairly poor population
					Houndé district (district hospital)
					Banfora district (regional hospital)
Group 3	0,65	42,43	6,66	61,20	Low utilisation; medium access; and very poor population
					Yako district (district hospital)
					Gaoua district (regional hospital)
Group 4	0,34	33,53	11,53	37,53	Very low utilisation; poor accessibility; fairly poor population
					Bogandé district (district hospital)
					Orodara district (district hospital)

### Data collection

Data collection was conducted from May to December 2012, using different tools, including structured questionnaires (1609 household interviews and 130 health worker surveys), medical records extraction (1752), key informant interviews (57, mainly in district and regional hospitals with facility in-charges, administrators, health workers in maternity and surgical wards, beneficiaries and in peripheral health centres with leaders and community representatives), and analysis of secondary data (from 1988 to 2010). Prior to data collection, all tools were pretested in Boromo District, which was not a study site, in October 2011 and January 2012. Table 2 gives an overview of the research methods used.

**Table 2:** Research methodsto evaluate the obstetric care subsidy policy in Burkina Faso, FemHealth project 2011–2013

Specific objective	Data	Timespan	Target population	Sample size
Method/data collection				
1. To determine if the introduction of the policy was followed by an increase of health services use, including facility-based delivery and caesarean section, and if the policy increased equity of access to health care				
Secondary data analysis of: -Routine data on health service deliveries and caesareans collected through annual health statistics reports published by the Ministry of Health; -Four Demographic & Health Survey datasetscombined to provide delivery-based trends in obstetric care over the period 1988–2010. The analysis focused on whether women delivered within a health facility and by caesarean. Sampling weights and clustering were taken into account in the analysis	MoH routine data: 1992, 1998, and 2000 to 2010; DHS data: 1993, 1998–99, 2003, 2010	1988–2010 (no data for 2004)	Routine data: national coverage DHS: Women of reproductive age (15–49 years) with at least one live birth in the five years preceding the survey	Routine data: national coverage DHS: 36,836 women
2. To analyze the costs incurred by the households during childbirth and collect the perception of people on the quality of services				
Structured household interviews with women who had just delivered or their relatives, on average 7 days after discharge. Interviews were performed by 9 experienced trained interviewers. All completed questionnaires were checked by a researcher before being sent for data entry.	Collected information included the socio-demographic characteristics of the women, the delivery events, the costs supported by the household as well as pre-referral costs, and women’s opinion about the health services they received.	From May to November 2012	Sampled women included: -All deliveries with near-miss complications or by Caesarean; -All deliveries with stillbirth, neonatal mortality or perinatal death under 7 days after birth occurring before discharge; -All deliveries with instrumental delivery or twins; - A sample of women with uncomplicated delivery which sub-sample size was indexed on that of the women with near-miss complication: half recruited in the hospitals and the remaining in health centers (one health center sampled per district)	A total of 1609 household interviews: 361, 165, 281, 235, 302, 265 in Banfora, Bogandé, Gaoua, Houndé, Orodara, and Yako, respectively, including 51, 52, 34, 41, 48, and 37from the six health centres
3. To evaluate the effects of the policy on the health system at district level (including both targeted and non-targeted health services), to examine potential changes induced by the introduction of the policy on the work patterns and motivation of health workers, and to assess the financing, financial effects on facilities and sustainability of the policy				
Data extraction from hospital registers and reports	Health District routine data and hospital register	Data extraction from 2005 to 2011	OPD clinics and Admission in the different unit (surgery, medicine, pediatric, OB/GYN), lengthof stay, lethality rate, human resources,	Outpatient and In Patient data of 6 hospitals
Semi-structured interviews with district key informants (health workers in maternity ward, block, peripheral health centres, administrators, and beneficiaries). Selected participants profile related to their involvement in the policy care provision in local health system and other community representatives and beneficiaries. Interviews were conducted by two experienced socio-anthropologists	Semi-structured interviews	From May to November 2012	Institutional leaders, administrators, health workers in maternity and surgical wards, beneficiaries (district and regional hospitals); staff head of units and community representatives (peripheral health centres)	57 semi-structured interviews
Structured interviews with health workers randomly sampled in all categories working in the hospital maternity, block or pediatrics wards (physicians, midwives, nurses, etc.), with the number of interviews per district weighted according to the size of the population of health workers in each district (number ranging from 16 in Bogandé to 29 in Orodara); interviews conducted by a sociologist.	Structured interviews	From October to December 2012	District health workers in maternity ward, block, peripheral health centres, administrators, and beneficiaries	130 structured interviews
Structured analysis of secondary financial data from national, district and facility levels	Extraction of financial data into spreadsheet	March- August 2012	Financial information systems at national level, in six study districts and selected facilities (13 in total).	National level, six districts, 1 university hospital, 2 regional hospitals, 4 district hospitals and 6 health centres.
4. To document the effect of the policy on severe maternal and neonatal morbidity and on quality of care				
Data extraction from the hospital medical records of the women (the same as for the household interviews above, but no extraction for women sampled in peripheral health centres) Data extraction performed by three trained health workers per hospital (including two in the maternity ward and one in pediatrics), using a standardized template. All completed extraction templates were checked by a researcher before being sent for data entry.	Medical records of the women and their babies	From May to November 2012	Data extraction for: -all womenwith near-miss with complications or C-section; -all women with stillbirth, neonatal mortality or perinatal death under 7 days after birth occurring before discharge; -all women with instrumental delivery or twins; -sample of women with uncomplicated delivery (sample size indexed on that of the women with near-miss complication): half recruited in the hospitals and the remaining in health centers (one health center sampled per district) - all women with maternal death.	1752 mothers and 182 infants.

### Data management and analysis

All completed questionnaires and templates were checked by supervisors and thereafter entered (using double entry) into an EPIDATA entrymask by two experienced data clerks. The database from the two data clerks was cross-checked for inconsistencies, in which case questionnaires were checked to ascertain the right information. The data analysis consisted of determining the mean, quartiles, minimum and maximum for cost data and determining the proportion for categorical data.

The data from the household interviews were analyzed according to the district, health care level, type of delivery (Caesarean, complicated, uncomplicated), and wealth quintiles. The wealth quintiles were created using the method reported by [13]. We used the most recent data available from the Demographic and Health Survey –DHS (http://www.measuredhs.com/). Common variables between DHS data and data from the structured interviews were identified, and a series of dichotomous variables (yes/no) were created for both the DHS and the interviews. A regression was performed using the DHS regression score from women’s data as the dependent variable and the variables of the identified goods as the independent variables. The correlation between the DHS score and the regression score was estimated as 0.978 (<0.001). The fitted values of the regression formed the basis of the weighted wealth quintiles and the cut points of the quintiles identified for use in the exit interview data. The analyses were performed using SPSS Version 20 software.

Data from four consecutive DHS (1993, 1998–99, 2003 and 2010) were combined to provide trends in utilisation of facility-based delivery care and caesarean sections over the period 1988–2010. The DHS collected information relating to all live births in the 5 years preceding the survey. In cases where a delivery resulted in a multiple birth, the place and mode of delivery was taken to be that of the latest-born infant. Segmented linear regression was used to calculate annual rates along with 95 % confidence intervals stratified by urban-rural or relative wealth, taking into account the complex survey design [14].

For the qualitative data the interviews with the key informant persons were recorded, transcribed and coded with N-Vivo software for thematic analysis [15]. A coding tree was elaborated prior to the analysis based on the research questions (deduction) and was reviewed and adapted with new codes during the analysis and the reading of the transcriptions (induction).

To assess the quality of care, we developed the concept of health care near-miss which measures the occurrence of omissions, delays and treatment failure [16, 17]. Health care near-misses were defined as negative events or omissions which occurred in the process of care but which did not necessarily lead to serious harm. One of our hypotheses was that a large increase of women reaching facilities could compromise safety and lead to medical errors. We calculated a score of omission (for vaginal delivery, Caesarean and care of the newborn) using between five and seven key clinical acts for each type of care. Thus, the omission score (Table 3) was calculated as the average of the proportion of women/new born for whom each key clinical act was not performed [16, 17]. The score of omission varied from zero (best quality) to seven (worst quality).

**Table 3:** Omission score and implementation score for vaginal and Caesarean deliveries and neonatal care in selected hospitals of Burkina Faso

Score of omission for :	Hospital	Mean score of omission (SE)	Median score of omission (IQR)	Score of policy implementation (rank of health facility in relation to offering the specified package at the right price)
Vaginal delivery	Houndé district hospital	0.41 (0.09)	0 (0, 1)	2
	Orodara district hospital	1.65 (0.14)	1 (1, 3)	1
	Banfora regional hospital	3.02 (0.12)	3 (2, 5)	5
	Gaoua regional hospital	3.09 (0.25)	5 (1, 5)	4
	Yako district hospital	2.22 (0.19)	2 (2, 3)	3
	Bogandé district hospital	3.33 (0.35)	4 (2, 5)	6
Caesarean	Houndé district hospital	1.51 (0.06)	1 (1, 2)	2
	Orodara district hospital	2.02 (0.02)	2 (2, 2)	1
	Banfora regional hospital	2.73 (0.07)	3 (3, 3)	3
	Gaoua regional hospital	2.93 (0.08)	3 (2, 4)	4
	Yako district hospital	3.61 (0.13)	4 (2.5, 4.5)	6
	Bogandé district hospital	4.07 (0.03)	4 (4, 4)	5
Neonatal care	Houndé district hospital	1.06 (0.21)	0 (0, 1)	-
	Orodara district hospital	3.37 (0.16)	4 (4, 4)	-
	Banfora regional hospital	2.37 (0.12)	2 (1, 4)	-
	Gaoua regional hospital	2.87 (0.20)	4 (1, 4)	-
	Yako district hospital	2.67 (0.26)	3 (0, 4)	-
	Bogandé district hospital	2.24 (0.35)	2 (0, 4)	-

*SE* standard error, *IQR* inter-quartile range

We also calculated a score of policy implementation (for uncomplicated, complicated, and caesarean deliveries) in order to assess if the quality of care was associated with the degree of policy implementation success (Table 3). This score was obtained by ranking hospital facilities based on the mean difference of the costs borne by households minus the theoretical amount they should have paid under the policy (taking into account other local initiatives for reducing fees). Items considered for estimating the total delivery cost to household included consultation, drugs and consumables, surgery (if any), transportation, payment for the delivery itself, delivery kit, surgery kit, post-operative kit, laboratory tests and ultrasonography, as well as any expenses borne outside the health facility, such as the purchase of drugs which were out-of-stock in the health facility pharmaceutical store. Theoretical amounts borne by households should have been USD22.26, USD7.29, and USD1.82 for a caesarean, a complicated delivery and a normal delivery, respectively (USD1 = CFA494.138, 2014 yearly conversion rate, http://www.oanda.com/currency/historical-rates/). All policy implementation scores for each type of delivery were then aggregated and a mean score for policy implementation was estimated and facilities ranked accordingly from one to six (Table 3). A lower score of policy implementation means that the mean costs borne by the household for delivery care were nearer to the official rate prescribed by the national subsidy policy.

Finally, we calculated a score for the availability of EmOC services and key service inputs (Table 4), which would help to better interpret the results for the quality of care [18]. A theoretical score was first calculated assuming a continual availability of services, personal and equipment. Thereafter, the score was adjusted taking into account the number of days where the operating theatres were not functional and the number of days of drugs stock-outs. A low score of availability of EmOC services and inputs is an indicator of lack of functionality (operating theatres not functional, absence of key personnel, absence of drugs and anesthetics etc). A low score could explain the poor implementation of the national policy, but *a contrario* poor implementation of the policy with a high score of EmOC services availability would highlight problems of non-compliance with the policy by the staff (frontlines workers and/or health district team).

**Table 4:** EmOCservices’ availability score in selected hospitals of Burkina Faso

Unit of analysis (hospital)	Bogandé	Yako	Orodara	Houndé	Gaoua	Banfora
Theoretical score of availability of services	14.0	13.0	14.0	13.0	14.0	13.0
Highest possible score = 17						
Theoretical score of availability for human resources	15.0	15.0	15.0	15.0	15.0	15.0
Highest possible score = 16						
Theoretical score of availability for material and drugs	14.4	8.2	11.9	7.7	8.2	12.4
Highest possible score = 33						
Weight: 1/1.94						
Total theoretical score of availability of EmOC services	43.4	36.2	40.9	35.7	37.2	40.4
Highest possible score = 50						
Weighting						
• Decrease of score because of non-functional operating theatre	0.5	0.3	0.0	1.2	0.0	0.0
• Decrease of score because of out of drug stocks	0.0	18.3	3.7	12.1	0.0	6.6
Corrected EmOC availability score	43.0	17.7	37.2	22.4	37.2	33.7

### Ethics approval and consent to participate

Ethical approvals were obtained from the national ethics committee for health research of Burkina Faso, the London School of Hygiene and Tropical Medicine (UK), and the Institute of Tropical Medicine Antwerp (Belgium). All participants gave written informed consent prior to interviews (for illiterate people, mainly for household interviews, consent was explained verbally in local languages before the form was signed).

## Results

### Roll out and financing of policy

The start of the policy to subsidize facility-based deliveries and EmOC was fixed by an official note for 1 October 2006 for EmOC in referral hospitals, and 1 January 2007 for normal deliveries in health centres. However, districts began on different dates, as shown by our key informant interviews: the referral hospitals of Banfora and Gaoua and district hospitals of Bogandé and Yakoin October-November 2006, the district hospital of Orodarain January 2007, the district hospital of Houndéin March/April 2007 and the health centres in January to April 2007.

According to our financial flows analysis, a government budget line provides funding for the subsidy, under the coordination of the Directorate for Family Health, which managed its implementation throughout. Resources were allocated initially according to a fixed amount per delivery type, using the annual estimate of the number of expected births, and later on the basis of the reimbursement of eligible expenditures actually incurred by health facilities. Over the first 5 years of the policy’s implementation, the budget was USD29.37 million, and disbursements to facilities over the same period totalled USD26 million. This equates to an average annual expenditure on emergency obstetric and neonatal care of about 2 % of total public health expenditure.

### Effects on utilisation of services

**Fig. 2:**
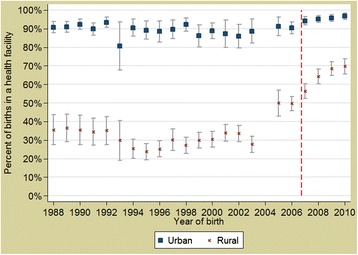
Annual trends of health facility deliveries in Burkina Faso, stratified by the residence. Footnote: *Red dashed line* represents the implementation of the obstetric care subsidy policy (2007)

**Fig. 3:**
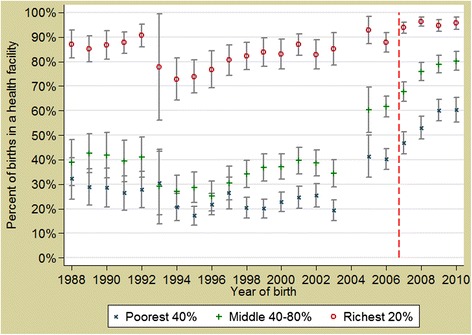
Annual trends of health facility deliveries in Burkina Faso, stratified by wealth quintile. *Red dashed line* represents the implementation of the obstetric care subsidy policy (2007)

Analysis of survey data indicates that deliveries in health facilities have increased since the policy started, especially in rural areas and amongst women from poor households (Figs. 2 & 3). During the period 1988 to 2010, there was an underlying secular trend of a 1 % increase in the facility-based delivery rate per year (*p* = 0.0157); after the policy was implemented, between 2007 and 2010, there was an additional 4 % increase in facility-based deliveries per year (*p* < 0.0001). The increase in facility-based delivery rates was greater in rural areas than urban areas (*p* < 0.0001) albeit from a lower base; and among poorer quintiles compared to wealthier groups (*p* < 0.0001).

**Fig. 4:**
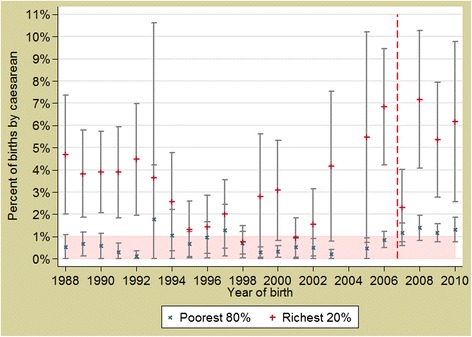
Annual trends of deliveries by caesarean in Burkina Faso, stratified by wealth. *Red dashed line* represents the implementation of the obstetric care subsidy policy (2007)

Between 1988 and 2010, there was no statistically significant trend in the caesarean rate per year (*p* = 0.7446) (Fig. 4); after the policy was implemented, between 2007 and 2010, the caesarean rate rose consistently above 1 % for the first time, however this increase was not statistically significant (*p* = 0.5588). There was no significant difference in trend according to wealth (*p* = 0.8882).

With respect to trends in non-targeted services such as in medical ward, surgical ward, paediatrics, and outpatients services, the subsidy had very little significant effect, though some cases of adverse effects were observed in our analysis of district-level routine health data, such as delays in routine services because of the demands of the targeted services and pressures from an overall lack of human resources.

### Effects on quality of care

Analysis found that the quality varied between hospitals, and did not appear to increase with hospital level, while the average cost borne by households in our exit interviews was significantly higher in a regional hospital than in a district hospital - respectively USD37.98 and USD25.72 (*p* < 0.0001). The absence of baseline data prior to policy implementation makes it impossible to attribute changes to the policy. However, hospitals with the highest level of implementation success of the subsidy (in other words, those for which patients’ fees were closest to the policy rates) are broadly those where the quality of care was also the highest (better management of deliveries and caesareans) and vice versa. This implies that the implementation of the policy is unlikely to have a notable negative effect on the quality of obstetric care.

The two hospitals with the highest omission scores for vaginal delivery (Gaoua and Bogande) (above 3 omissions) also had high frequency of readmission in the delivery room for retained placenta (above 10 %); and case fatality rates among women with severe obstetric complications were highest in the two regional hospitals and Bogande and Yako district hospitals, where omission scores for caesareans were also high (Table 5). Omission scores for neonatal care are similar across facilities (around 2–3), except for Hounde district hospital (around 1), which also performs very well for vaginal and caesarean deliveries omissions scores.

**Table 5:** Readmissions rate in delivery room after uncomplicated delivery and fatality rate among women with severe obstetric complication in selected hospitals of Burkina Faso

Hospital	Readmission in delivery room (%)	Fatality rate among women with severe obstetric complication (%)
Houndé district hospital	0	0
Orodara district hospital	0.91	1.28
Banfora regional hospital	2.67	4.92
Gaoua regional hospital	10.53	10.14
Yako district hospital	3.70	1.61
Bogandé district hospital	11.54	6.45

Overall, 40 % of maternal near-misses were from Banfora district. Most (75 %) of the maternal near-misses were referred from another health facility. In addition, 78.6 % of maternal deaths were observed in the two regional hospitals of Banfora and Gaoua.

### Effects on household costs

The amounts reported by households in exit interviews have decreased significantly, in comparison with amounts found in studies conducted shortly before the introduction of the policy to subsidize facility-based deliveries and EmOC (Table 6). The mean cost before 2006 for hospital delivery from a cohort of 1014 women sampled from three district hospitals, two regional hospitals, and two university hospitals was USD55.14. That is more than twice the mean cost observed in the present study -approximately USD26.53 [19]. However, the amounts paid for health services, primarily in hospitals (average cost between USD13.69 and USD29.35), are much higher than the rates set by the policy. Some costs which should be covered by the policy have often been borne by the households (transport from peripheral health centre to hospital, drugs, laboratory tests, and informal payments).

**Table 6:** Average and median delivery costs (in USD) paid by the households in Burkina Faso and scores of implementation, by type of delivery and health facility

Hospital	Uncomplicated delivery	Caesarean	Complicated delivery	Mean cost per delivery
Houndé district hospital	4.65 (1.82) [2]	32.58 (28.33) [2]	12.33 (9.71) [3]	16.52 (13.29) [2]
Orodara district hospital	4.46 (1.82) [1]	26.05 (22.26) [1]	10.56 (7.89) [1]	13.69 (10.66) [1]
Banfora regional hospital	19.37 (21.73) [5]	40.42 (34.81) [3]	23.28 (19.72) [6]	27.69 (25.42) [5]
Gaoua regional hospital	16.45 (8.40) [4]	41.32 (33.80) [4]	21.62 (16.59) [5]	26.46 (19.60) [4]
Yako district hospital	13.78 (6.47) [3]	46.21 (38.15) [6]	11.16 (7.69) [2]	23.72 (17.43) [3]
Bogandé district hospital	-	44.17 (40.07) [5]	14.53 (10.32) [4]	29.35 (25.20) [6]
CSPS (health centre)	2.72 (1.82) [NA]	-	-	2.72 (1.82) [NA]

Median delivery costs in brackets; scores of implementation in square brackets

In Bogandé, Orodara and Yako, other financing sources provide additional funds to cover partially (Orodara and Yako) or totally (Bogandé) the 20 % of caesarean cost to be borne by the users. Thus women with caesareans in these three districts should have paid 0, USD10.12, and USD8.9 in Bogandé, Orodara and Yako respectively. The mean cost supported by the households in Orodara was lower than in all other districts. However, in Bogande and Yako, where similar cost sharing initiatives operate, no reduction in household costs was found.

Households continue to find it difficult to meet the costs associated with childbirth: 7 % reported that they were unable to pay and in rare cases (1.4 % overall, with the higher rate [4.6 %] observed in Gaoua), women have had to discharge themselves from hospital early on economic grounds. Only 2.2 % of women had health insurance; the great majority of them were from Orodara (6.6 %) and Houndé districts (3 %). There were no reports of households taking advantage of the policy’s provision for the indigent. Two thirds (63 %) of interviewed women were living in rural areas (a distribution quite comparable to that reported for the DHS 2010 sample), but 57.2 % of the sample included women living in households classified in the two richer quintiles (4 and 5), compared to only 24.2 % from the poorer households (1 and 2). Apparently, women from less poor households are those who are most able to reach the higher levels of the health system, in search of more skilled care, indicating that financial and other barriers persist.

### Awareness and user satisfaction

The mean proportion of women or their relatives who were aware of the policy was low: 52 % on average and varying from 84 % in Yako district to 20 % in Houndé district. Respondents reported their overall satisfaction with the services they received (waiting times, quality of treatment received, costs of care, availability of drugs etc.). However, in Banfora there was significant dissatisfaction with the cost (5 %) and the availability of drugs (23 %). In addition, dissatisfaction has also been reported in relation to the cleanliness of premises (9 %), mainly at Orodara (20 %) and Gaoua (13 %).

### Effects on staff

**Fig. 5:**
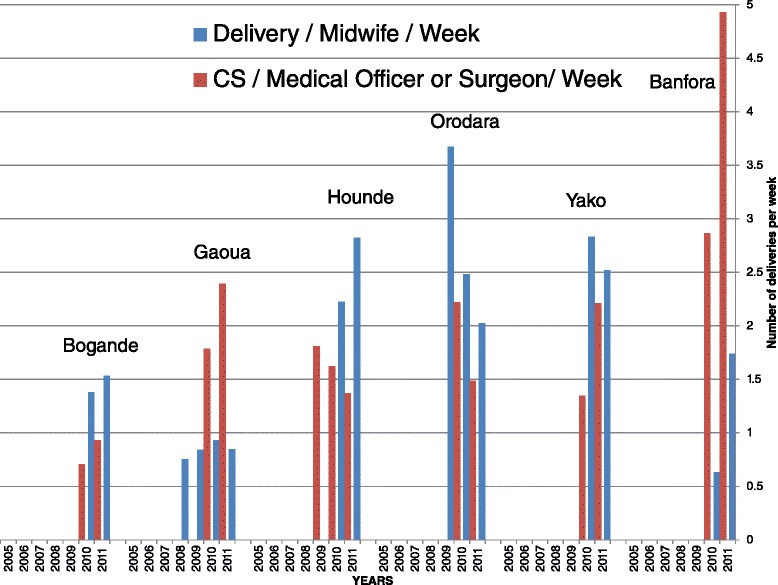
Trend in the number of deliveries per week, by type of health worker from 2005 to 2011

With respect to the motivation of health personnel, the workers frequently reported an increased workload resulting from policy implementation (not just clinical but also administrative in order to comply with policy reporting requirements), and that they did not gain any direct financial benefit in compensation. However, the majority of workers exhibited good motivation, in part because of the improvement of working conditions made possible through the implementation of the policy. For example, seven out of ten health staff reported higher work motivation, partly because the policy enabled them to provide more timely and adequate care. Indirectly, the increased facility-based deliveries may have raised health personnel revenue: 41 % reported an increase in their revenue sharing over the period. Analysis of routine data on staff to workload ratios by district suggests varying patterns of change and that overall the workload remains acceptable and manageable (Fig. 5). It did not exceed four deliveries per week on average for midwives and five caesareans per week on average for physicians.

### Effects on facilities and interactions with the health system

Financial analysis and interviews with staff and managers suggest that the policy has resulted in greater flexibility for facilities, by making available more financial resources and medicines. Resources were made available throughout six-monthly reimbursements of the health facilities, based on the estimated amount they incurred in treating women. However, interviews also revealed constraints that hamper the implementation of the policy, such as gaps in continuity and quality of the services (e.g. a lack of ambulances for referrals).

“…*The main concern is about the transportation for the implementation of SONU* [the policy] *because for a lady if we declare that there are complications, we must bring her to the CMA* [the district hospital]*. If there is not a functional ambulance, it poses a problem*” (administrative respondent)

Interviews suggest that cases of unsuccessful implementation were related to institutional constraints and non-compliant practices by some health workers, especially in Orodora district. These problems resulted from insufficient qualified staff, inadequate equipment or stock-outs of inputs (e.g. non-availability of tests or medicines in the facilities’ pharmacy), obliging users to pay for external services. Non-compliant practices refers mainly to unscrupulous service providers in hospitals, who take advantage of the ignorance of users about the policy to claim full payment for procedures, or to stockpile medicines through fake prescriptions in order to illegally resell them subsequently to unaware users.

“…*these kits were a source of enrichment for a certain category of persons*” (health staff respondent)

### Understanding variable implementation of the policy

As well as analysing effects, the FEMHealth team used in-depth interviews and analysis of secondary data to understand why implementation of the policy varied across districts, using a realist evaluation approach [7]. It seems that one factor was the lack of clarity in the official guidelines of the policy, which negatively impacted on its implementation (e.g. unclear inclusion of associated pathologies or lack of precise definition of certain procedures). The adoption of the policy by the local health systems was also done with both positive and negative changes. Positive adaptation included the arrangements made in all districts to better control the use of medicines by care providers, following various non-compliant practices. There was also the pre-financing, especially in health centres, of the costs of medicines to maintain service delivery, thus overcoming delays due to reimbursement procedures. Negative adaptation included non-compliant practices (e.g. the “dubious” management of kits, prescription without real pathologies and the sale of leftover medicines to users, all for personal gains).

## Discussion

The study’s findings are broadly consistent with wider literature, which highlight the potential of fee exemptions to improve uptake of maternal and other health care services and to improve equity, but only when properly implemented and accompanied by the necessary resources to fund reimbursements to providers as well as wider investments in strong health systems [9, 20, 21].

The concomitance of the change of the trends in the facility-based births in some socioeconomic groups (Figs. 2 & 3) with the policy implementation leads to a strong presumption that the observed increase stems from the impact of the subsidy policy. De Allegri et al. [22] who conducted a household survey in Nouna District arrived at similar conclusions about the subsidy policy impact in increasing the proportion of deliveries taking place in health facilities. However, for caesareans, where the change in trends started before the introduction of the policy, other factors might have contributed too, for example the building of several district hospitals and training of general physicians in EmOC. Analysis from a wide range of countries finds that women from richer households tend to benefit more from caesarean deliveries, as result of higher access, awareness and ability to pay [23]. In the Burkina context, some households could not afford even a subsidized caesarean (costing USD22.26, plus transport costs).

Although the indirect finding that the quality of care did not appear to suffer because of the increased uptake of services is positive, we were expecting that the quality of care (as measured by omission scores) would be better in the secondary referral (regional hospital) level than in the primary referral (district hospital) level, because of the presence of more qualified health personnel. However, this was not systematically the case, especially with respect to vaginal birth omission scores. Regional hospitals have a greater focus on complicated and surgical deliveries, for which they performed slightly better with respect to omission scores (but still not as well as some district hospitals). Delays in accessing referral care, as well as poor case management throughout the health system, may be two of the contributing factors to the higher volume of maternal deaths and near-misses (75 % of maternal deaths from all the six districts were found in the two regional hospitals) and high case fatality rates.

The policy has reduced the cost supported by the households for facility-based deliveries, but unfortunately, it has not yet enabled fully equitable access to emergency care for poor families [19, 24–26]. It is likely that the application of the provision of the policy on indigents would further reduce the inequities of access – this policy has yet to be put into real practice [4, 11, 27, 28].

Our findings corroborate and add to those from previous studies conducted in Burkina Faso on the EmOC subsidy policy, particularly the findings related to:

- the reduction of costs of facility-based deliveries, which however remain higher than they should have been under the policy [25, 26];
- the increase of service utilization [22];
- the acceptable level of health workers’ workload despite the increase in service utilization [29];
- the identification of constraints in the local health systems which could affect the outcome of the policy implementation, such as the availability of equipment, drugs and consumables [4, 26];
- the lack of exemption for indigent people [4, 28];
- the importance of local health system context to successfully implement the policy [26].

The strengths of this study included:

- Combining several methods using a wide range of tools, making triangulations across methods possible, and combining qualitative and quantitative approaches [7];
- Taking into account several levels of the health care system (the previous studies were focused on the district level);
- The robustness of the secondary data analysis, which was country-wide, involved data from two sources (routine collection and national surveys), and covered a long period (from 1988);
- Collecting prospective socioeconomic and medical data for women from five out of 13 regions.
- Building objective indicators to measure the functionality and quality of services and relating these to policy implementation;
- Proactively assessing unintended effects on some tracer services (outpatients, as well as surgical, medical and paediatric cases);
- Using realist evaluation techniques to probe not only what happened but also why.

Some general study limitations are highlighted here, including incompleteness of routine data at district and central levels, the lack of baseline data for several key indicators of impact of the policy, and the absence of any control areas. In addition, each tool had its own constraints, which are discussed in more detail in the country protocol and report [10, 11]. The findings are not necessarily generalisable beyond the study areas; however, we have identified some of the contextual factors which contribute to patterns of implementation and results, which help readers to interpret the implications of findings for other settings.

## Conclusions

The FEMHealth study in Burkina Faso adds to the growing body of international evidence on the costs and effects of policies to reduce financial barriers to obstetric care, and includes elements which are rarely incorporated in evaluations, such as actively seeking possible unintended effects and looking at systems effects of policies and determinants of implementation in different sites.

Our evidence suggests that the implementation of the national policy to subsidize facility-based deliveries and EmOC helped to improve the delivery of services by health care facilities. The policy has also helped to reduce the costs paid by households, even though they remain higher than expected. These findings highlight the importance of maintaining the subsidy policy, given the evidence of positive outcomes, but they also point out areas where attention is needed to ensure the poor and most vulnerable population benefit fully from the policy. The findings suggest there is a need to focus on equity in the provision of quality care, in order to facilitate access to healthcare for the poorest people at all levels of the health system. They also emphasize the need for greater attention to good governance in facilities, without which success is not possible, even when inputs and services are available.

A number of recommendations emerge from the study, including the need to strengthen the knowledge and skills of the health personnel (highlighted by the quality of care scores) and improve equipment (also reflected in sometimes poor functionality scores) for better quality of care of newborns in accordance with the guidelines of the subsidy policy. The budget line to cover all costs for indigents must also be put into effect to overcome the persistent barriers to access to care faced by the poorest groups (this is raised by the equity analysis as well as the financial analysis). At facility level, more effective controls should be established to stop non-compliant practices and avoid additional costs for users, which were raised in our exit interviews and key informant interviews. Best practices in the management of kits should be established and disseminated to rationalize the use of drugs. Guidelines for the policy should be clarified and the administrative workload for staff reduced, as requested by staff and managers interviewed.

## Availability of data and materials

Further reports and supporting materials can be found at www.abdn.ac.uk/femhealth.

## Abbreviations

EmOC: emergency obstetric care
DHS: demographic and health survey
USD: United States Dollar
